# Effect of addition of chitosan to self-etching primer: antibacterial activity and push-out bond strength to radicular dentin

**DOI:** 10.7555/JBR.26.20120042

**Published:** 2012-07-06

**Authors:** Shaymaa Elsaka, Amr Elnaghy

**Affiliations:** a Department of Dental Biomaterials, Faculty of Dentistry, Mansoura University, Mansoura, PC 35516, Egypt;; b Department of Conservative Dentistry and Endodontics, Faculty of Dentistry, Mansoura University, Mansoura, PC 35516, Egypt.

**Keywords:** *Enterococcus faecalis*, antibacterial activity, chitosan, push-out, primer, radicular dentin

## Abstract

The purpose of this study was to evaluate the antibacterial activity of a modified self-etching primer incorporating chitosan and whether this modification affected the bond strength to radicular dentin. A modified self-etching primer was prepared by adding chitosan solutions at 0.03%, 0.06%, 0.12% and 0.25% (*W/W*) to RealSeal selfe-tching primer. RealSeal primer without chitosan was used as the control. The antibacterial activity of the modified self-etching primer was evaluated using the direct contact test against *Enterococcus faecalis*. The bonding ability of the RealSeal system to radicular dentin was evaluated using the push-out bond strength test. The modes of failure were examined under a stereomicroscope. Data were analyzed using analysis of variance (ANOVA) and Tukey's test, with a *P*-value < 0.05 indicating statistical significance. The results showed that the antibacterial properties of the freshly prepared and aged modified self-etching primer incorporating chitosan exhibited potent antibacterial effect against *Enterococcus faecalis* compared with the unmodified primer. The RealSeal system with the aged modified self-etching primer incorporating chitosan showed no significant differences in the bond strength as compared with the control (*P* = 0.99). The findings suggest that modified self-etching primer incorporating chitosan is a promising antibacterial primer which does not adversely affect the bond strength of the RealSeal system to radicular dentin.

## INTRODUCTION

One of the main goals of endodontic treatment is to eliminate bacteria from the root canal system and prevent subsequent reinfection[1]. *Enterococcus faecalis* (*E. faecalis*) is the most common and frequently the only species to persist in endodontically treated teeth[2],[3]. This highlights the ability of *E. faecalis* to resist endodontic disinfectants and survive nutrientstringent conditions within root-filled teeth[2]. A possible treatment modality is the use of root canal sealer with antibacterial properties to improve the outcome of endodontic treatment[4]–[6].

In recent years, methacrylate resin-based sealers have been developed on the basis of dentin adhesion technologies in an attempt to seal the root canal more efficiently and strengthen the root structure[7]–[10]. Long-term sealing ability and close adaptation to the root canal walls are one of the prime fundamentals for a root canal sealer[6],[10]. RealSeal (SybronEndo, Orange, CA, USA) has been introduced as an alternative to gutta-percha and conventional sealers. The RealSeal system includes primer, sealer, and core material[11],[12], which form a socalled monoblock between the canal wall, sealer, and RealSeal cone[13]. RealSeal sealer contains urethane dimethacrylate, polyethylene glycol dimethacrylate, ethoxylated bisphenol A dimethacrylate, and bisphenol A glycidyl methacrylate resins, silane-treated barium borosilicate glass, barium sulfate, silica, calcium hydroxide, bismuth oxychloride with amines, peroxide, photo initiator, and pigments as well. The self-etching primer consists of hydroxyethyl methacrylate, sulfonic acid and water. RealSeal core material contains polyester polymer polycaprolactone, bioactive glass and radiopaque fillers. The RealSeal sealer is a dualcure, resin-based composite sealer[14]. The new system has been reported to exhibit excellent sealing ability[15]; however, it has no antibacterial properties[16]–[18]. Therefore, therapeutic benefit may be gained when combining an antibacterial agent with this system.

Chitosan is a naturally occurring polysaccharide biopolymer that is obtained by alkaline partial deacetylation of chitin. Chitin is a straight homopolymer consisting of (1,4)-linked *N*-acetyl-glucosamine units, which is found in the exoskeleton of crustaceans such as crabs and shrimps. Chitosan is a copolymer composed of glucosamine and *N*-acetyl-glucosamine. The antibacterial property of chitosan is related partly to the interaction between positively charged chitosan and negatively charged bacterial cell surface that would decrease bacterial cell permeability, resulting in cell death. Chitosan is generally regarded as biocompatible, non-toxic, biodegradable, and is inherently antibacterial in nature[19],[20]. In recent years, the use of chitosan has become a significant area of research in dentistry[21]–[23]. The aim of this study was to investigate the antibacterial activity of a modified self-etching primer incorporating chitosan and the outcome of this modification on the bond strength of RealSeal system with the modified primer to radicular dentin. The null hypothesis was proposed that when using a direct contact test with *E. faecalis* and a push-out test with endodontically prepared single-rooted permanent teeth, additional incorporation of chitosan into a self-etching primer of the RealSeal system resulted in no significant differences in antibacterial activity and adhesive bond strength to radicular dentin after seven days.

## MATERIALS AND METHODS

### Reagents

RealSeal self-etching primer (Lot number: 182766; SybronEndo, Orange, CA, USA) was used in this study. A modified self-etching primer was prepared by adding chitosan solution (85% deacetylated; Sigma-Aldrich Chemical Co., St Louis, MO, USA) at 0.03%, 0.06%, 0.12% and 0.25% (*W/W*) to RealSeal self-etching primer. Chitosan solution was prepared by dissolving 2 g of chitosan powder in 1 L of 1% (*V/V*) acetic acid[23]. RealSeal self-etching primer without chitosan solution served as the control.

### Test microorganism and growth conditions

*E. faecalis* (ATCC 29212) was obtained from the Department of Microbiology, Faculty of Medicine, Mansoura University, Mansoura, Egypt. *E. faecalis* was cultured in brain heart infusion (BHI) broth (Oxoid, Hampshire, England) overnight at 37°C.

### Direct contact test (DCT)

The DCT is based on the turbidometric determination of bacterial growth in 96-well microtiter plates (Becton, Dickinson and Company, Franklin Lakes, NJ, USA)[24]. Briefly, a sterile 96-microtiter plate was held vertically, and the sidewalls of 8 wells were coated with 15 µL of each tested modified self-etching primer. The primer was handled according to the manufacturer's instructions. Then, a 10 µL bacteria suspension (1×10^6^ CFU/mL) of *E. faecalis* was placed on the tested material for 1 h at 37°C. Afterwards, the BHI broth (235 µL) was added to each well and gently mixed (DELFIA® Plateshake; PerkinElmer Inc., Boston, MA, USA) for 2 min. The positive control consisted of a set of 8 uncoated wells in the same microtiter plate containing the bacterial inoculums, which was processed as described previously; while the negative control consisted of a set of 8 wells coated with the test materials containing an equal volume of non-inoculated fresh medium. The antibacterial properties of the modified self-etching primers were assessed 1 h after application (fresh preparations). Similar experiments were conducted when the modified tested self-etching primers were aged in 280 µL of phosphate-buffered saline (PBS) (Sigma-Aldrich) for 7 d at 37°C before assaying. During the aging period, PBS was replaced every 24 h. The kinetics of bacterial growth in each well was measured every 20 min for 16 h at 650 nm by using a temperature-controlled spectrophotometer (VICTOR® X Multi-label Plate Readers; PerkinElmer Inc., Boston, MA, USA) at 37°C. Auto-mixing was done before each reading to establish a homogeneous bacterial cell suspension. Data were recorded in optical density units. The baseline represented the values obtained from the negative control wells, and was then subtracted from the respective experimental data, and plotted on growth curves.

### Specimen preparation

Fifty extracted, single-rooted human teeth were used in this study. The study was reviewed and approved by the institutional review board of the Faculty of Medicine and Dentistry, Mansoura University, Mansoura, Egypt. All the teeth were radiographed to ensure the presence of a single canal. Only teeth that were free of cracks examined under a stereomicroscope (Olympus SZX-ILLB100; Olympus Optical Co., Ltd., Tokyo, Japan) at 10× magnification were used. The teeth were placed in sodium hypochlorite (NaOCl) for 2 h for surface disinfection and periodontal ligament removal followed by storage in distilled water until use.

The access preparation was done with a high speed #4 round bur and the pulp tissue was removed with a barbed broach. For each tooth, canal patency was established using a #10 Flex-o-file (Dentsply Maillefer; Tulsa, OK, USA). The root canals were enlarged with the ProFile nickel-titanium rotary instruments (Dentsply Maillefer; Tulsa, OK, USA) using the crown-down technique. Each canal was prepared to ISO size 30, 0.06 taper and the working length was established 1 mm short of the apex. The root canals were irrigated with 3% NaOCl between instruments using a 27-gauge Monoject endodontic needle (Sherwood Medical, St. Louis, MO, USA). Then, the canals were irrigated with 17% ethylenediaminetetraacetic acid (EDTA) and 3% NaOCl. Finally, the roots were irrigated with 10 mL of distilled water to avoid the prolonged effect of EDTA and NaOCl solutions. The debrided root canals were dried with multiple paper points and divided into five groups, of 10 each group, on the basis of chitosan solution incorporation in the modified self-etching primer.

### Canal filling

The modified RealSeal self-etching primer was introduced into the canal with a microbrush and allowed to remain for 30 s, and the excess was removed with dry paper points. RealSeal sealer was then introduced into the canal space using a Lentulo spiral instrument (Dentsply Maillefer, Ballaigues, Switzerland). A size-30 Resilon point, previously tried-in with tug back, was coated with RealSeal sealer and inserted to the working length. Afterwards, the Resilon point was compacted using a warm vertical compaction technique with a System B (SybronEndo, Orange, CA, USA) at 150°C. Backfilling was achieved with Obtura II (Spartan; Fenton, MO, USA) at 140°C. Then, the coronal surface of the root filling was light-cured for 40 s with a Coltolux LED curing light (light intensity: 1200 mW/cm^2^; wavelength: 450-490 nm; Coltene Whaledent Product, Cuyahoga Falls, OH, USA) to accomplish an immediate coronal seal. All access cavities were sealed with Cavit (3M ESPE; Seefeld, Germany). The specimens were stored at 37°C and 100% humidity for 1 week to allow the sealer to set completely.

### Push-out assessment

Each root was horizontally sectioned into five slices of 1.0±0.1 mm thick by using a low-speed diamond saw (Isomet 1000; Beuhler Ltd., Lake Bluff, IL, USA) under water giving a total of 50 specimens for each group. Specimens containing filling material of a noncircular shape were discarded, as this would result in non-uniform stress distributions during testing and inaccurate results[25]. After the thickness of each slice was measured with a digital caliper (Mitutoyo, Tokyo, Japan), the filling material was loaded with a 0.5-mm diameter cylindrical plunger[26]. Loading was performed on a universal testing machine (Model TTB; Instron Corp., Canton, MA, USA) at a cross-head speed of 0.5 mm/min until debonding occurred. The load at failure recorded in newtons (N) was divided by the area of the bonded interface for each specimen to calculate bond strength in megapascals (MPa) using the following formula[27]: 
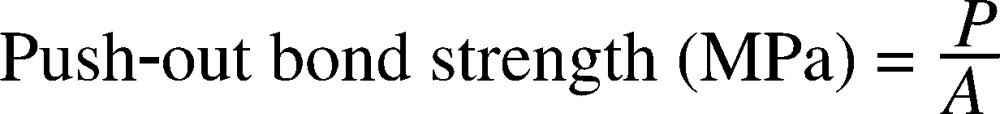


Where *P* is the maximum load (N), and *A* is the adhesion area of root canal filling (mm^2^). The adhesion area of each section was calculated by using the following formula: 
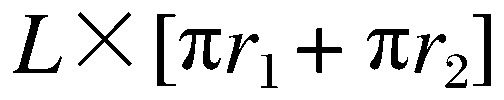

*L* was calculated as follows: 
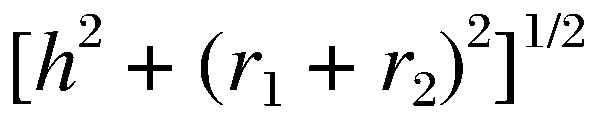


where *r*_2_ is the coronal radius, *r*_1_ is the apical radius, and *h* is the thickness of the slice.

Debonded specimens were examined under a stereomicroscope at 50× magnification to evaluate the fracture pattern. The modes of failure were classified as adhesive failure along the sealerdentin interface, cohesive failure within the sealer or mixed failure.

### Statistical analysis

Data were presented as mean±standard deviation (SD). The data for bacterial growth rate of antibacterial activity and push-out bond strength were analyzed using one-way analysis of variance (ANOVA) and Tukey's multiple comparison tests. A *P*-value < 0.05 was considered statistically significant.

**Fig. 1:**
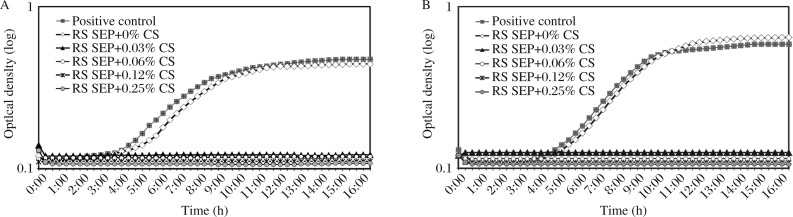
*Enterococcus faecalis* growth after direct contact with fresh material (A) and material aged for 7 d in phosphate-buffered saline (B). Each point on the curve is the average optical density on a logarithmic scale measured in 8 separate wells at the same time Each point on the curve is the average optical density on a logarithmic scale measured in 8 separate wells at the same time. CS: chitosan; RS SEP: RealSeal self-etching primer.

## RESULTS

### Antibacterial activity

The growth curves for all the freshly tested specimens showed antibacterial activity of the modified RealSeal self-etching primer incorporating 0.03%, 0.06%, 0.12% and 0.25% (*W/W*) chitosan solutions (***Fig. 1A***). The bacterial growth of the modified RealSeal self-etching primer incorporating chitosan solution was significantly reduced as compared with the control (*P* < 0.001, *F* = 84.878) (***Table 1***). After aging of the materials for 7 d, the modified RealSeal self-etching primer incorporating 0.03%, 0.06%, 0.12% and 0.25% (*w/w*) chitosan solutions maintained their antibacterial properties (*P* < 0.001, *F* = 269.132) (***Fig. 1B***, ***Table 1***).

### Push-out bond strength

The mean push-out bond strength (MPa), standard deviation (SD), number of specimens (n) and the percentage (%) of failure modes of all groups are presented in ***Table 2***. There was no significant difference in the bond strength between the RealSeal system with modified RealSeal self-etching primer incorporating chitosan solution and the control group (*P* = 0.99, *F* = 0.064). Most failure modes were adhesive failures in all groups (***Table 2***).

## DISCUSSION

The antibacterial activity of the root canal filling materials and sealers may help eradicate the remaining microorganisms, which are not affected by the chemomechanical preparation or intracanal medication, and it may also suppress infection[1],[28]. Therefore, to avoid the growth of bacterial attachments, the root canal filling materials should have an antibacterial effect if possible[16].

*E. faecalis* was chosen as it is commonly found in infected root canals[28],[29], which is the most frequently isolated microorganism in re-treated cases of apical periodontitis[30], with an incidence ranging from 24% to 77%. This finding is due to various virulence factors, including its ability to oppose other microorganisms, invade deeply into dentinal tubules and survive nutritional deficiency[31]. Consequently, antibacterial activity against *E. faecalis* is important to clinical practice and, accordingly, this microorganism was chosen for the present study.

The DCT was developed to determine the effect of direct contact between the tested materials and a monolayer of the tested microorganism[24]. DCT is a quantitative method and provides information on the viability and bacterial growth rate. According to the results of the current study, for the unmodified self-etching primer (RealSeal self-etching primer + 0% chitosan solution), the set specimens did not produce bacterial growth inhibition (***Fig. 1*** and ***Table 1***). On the other hand, the modified self-etching primer incorporating chitosan solution exhibited a bacteriostatic effect, demonstrating inhibition of bacterial growth compared with the control primer (RealSeal self-etching primer + 0% chitosan solution) for both fresh and aged specimens. This could be explained by the polycationic properties of chitosan solution, formed by the positively charged -NH_3_^+^ groups of glucosamine, which could be the main factor contributing to its interaction with negatively charged surface components of bacteria, resulting in extensive cell surface alterations, leakage of intracellular contents and ultimately causing damage to vital bacterial activities[32],[33]. Accordingly, it is possible that the RealSeal self-etching primer incorporating chitosan solution inhibits the growth of the invading bacteria and subsequently inhibits bacterial re-entry and re-colonization.

**Table 1:** Mean±SD of the bacterial growth rate as reflected by the slope of the linear portion of the growth curve* and Tukey's analysis

Group	Fresh material (1 h)	Aged material (7 d)
Positive control	0.92±0.08^a^	4.27±0.29^a^
RS SEP + 0% CS	0.89±0.06^a^	4.65±0.30^a^
RS SEP + 0.03% CS	0.52±0.04^b^	1.49±0.18^b^
RS SEP + 0.06% CS	0.50±0.03^b^	1.40±0.16^b^
RS SEP + 0.12% CS	0.47±0.03^b^	1.28±0.17^b^
RS SEP + 0.25% CS	0.45±0.03^b^	1.20±0.15^b^

*Each value in the table is the average optical density (×10^−2^) ± SD (×10^−3^) of the slope of bacterial growth in 8 separate wells in the same microtiter plate. Mean values with the same superscript lowercase letter (column) are not significantly different (*P* > 0.05). CS: chitosan; RS SEP: RealSeal self-etching primer.

**Table 2:** Mean 7 d push-out bond strength (MPa) value, standard deviation (SD), number of specimens (*n*), and percentage (%) distribution of failure modes of debonded specimens, in each group.

Group	Mean±SD	*n*	Mode of failure (%)		
			Adhesive	Cohesive	Mixed
RS SEP + 0% CS	0.54±0.29	50	50	2	48
RS SEP + 0.03% CS	0.53±0.22	50	58	0	42
RS SEP + 0.06% CS	0.51±0.26	50	54	0	46
RS SEP + 0.12% CS	0.50±0.25	50	56	0	44
RS SEP + 0.25% CS	0.47±0.25	50	60	0	40

CS: chitosan; RS SEP: RealSeal self-etching primer.

Even though the root canal sealers may possess new biological functions, these would not be clinically useful if the original sealer properties are hampered by the addition of such new characteristics. The bond strength of the root canal sealers to radicular dentin is important for maintaining the integrity of the seal in root canal filling[34],[35]. Optimum adhesion requires intimate contact between the adhesive material and the substrate to facilitate molecular interaction and allow either chemical adhesion or penetration for micromechanical surface interlocking[36]. Adhesion of an endodontic sealer is defined as its capacity to adhere to the root canal walls and the ability to promote the union of the gutta-percha cones to each other and to dentin[37],[38]. This concept can be applied to the filling systems that use different solid materials combined with the root canal sealer[37]. The bond strength of the root canal sealers to radicular dentin is essential for preserving the integrity of the seal in the root canal filling[39]. The recent introduction of the RealSeal system as an alternative root canal filling material is based on the formation of a so-called single resin block (monoblock), in which the core material, sealing agent and root canal dentin form a single cohesive unit that adheres to the root canal walls[40].

Bond-strength testing has become a popular method for determining the effectiveness of adhesion between the endodontic materials and tooth structure. There are various methods for measuring the adhesion of endodontic root canal sealers; however, none has yet been widely accepted[40],[41]. The tensile strength test is sensitive as small alterations in the specimen or in stress distribution during load application have a substantial influence on the results[42]. On the other hand, a major problem with the shear testing is that it is difficult to closely align the shear-loading device with the adhesive interface. The load is offset at some distance from the bonded interface, resulting in unpredictable torque loading on the specimen[43].

The push-out test is a reliable and efficient method to evaluate the bond strength because it allows measurement of regional differences in bond strength along the root length with adequate variability of the data distribution[39]. Another advantage of this method is that it allows the root canal sealers to be evaluated even when bond strengths are low[40]. Accordingly, the bonding ability of the RealSeal system with modified self-etching primer incorporating chitosan solution to radicular dentin was evaluated using the push-out bond strength test in the present study.

Incorporation of chitosan solution into RealSeal self-etching primer was found to be advantageous, since the bond strength of RealSeal system with modified self-etching primer incorporating chitosan solution to radicular dentin was not significantly different from that of the control. Interestingly, the fracture mode analysis showed that the adhesive failure across the bonding interface was the most common mode in all groups.

This study highlighted the possible potential consideration of chitosan solution as a significant component in the RealSeal self-etching primer to ensure long-term success of endodontic treatment, suggesting a promising usage of chitosan solution in resisting bacterial growth at the radicular dentin-resin interface. Longer aging experiments as well as clinical evaluation will be carried out to determine the most efficient concentration of chitosan solution in future studies. The results of this study rejected the null hypothesis since the incorporation of increasing concentrations of chitosan solution into RealSeal self-etching primer improved its antibacterial activity against *E. faecalis* without affecting the bond strength of RealSeal system to radicular dentin.

In conclusion, based on the results presented and within the limitations of this *in vitro* study, it is concluded that the RealSeal self-etching primer incorporating 0.03%–0.25% (*W/W*) chitosan solution exhibits remarkable 7-day antibacterial activity against *E. faecalis* compared with the unmodified primer, and incorporation of chitosan into RealSeal self-etching primer does not affect significantly the push-out bond strength of the RealSeal system to radicular dentin.
